# Acetone as biomarker for ketosis buildup capability - a study in healthy individuals under combined high fat and starvation diets

**DOI:** 10.1186/s12937-015-0028-x

**Published:** 2015-04-22

**Authors:** Amlendu Prabhakar, Ashley Quach, Haojiong Zhang, Mirna Terrera, David Jackemeyer, Xiaojun Xian, Francis Tsow, Nongjian Tao, Erica S Forzani

**Affiliations:** 1 Current address: Center for Bioelectronics and Biosensors, the Biodesign Institute, Arizona State University, 1001 S McAllister Ave., Tempe, AZ 85287 USA; 2 School for Engineering of Matter, Transport, and Energy, Arizona State University, 501 E. Tyler Mall, Tempe, AZ 85287 USA; 3 School of Electrical, Computer, and Energy Engineering, Arizona State University, 650 E. Tyler Mall, Tempe, AZ 85281 USA

**Keywords:** Ketogenic diet, Breath ketone, Acetone, Fasting ketosis, Nutritional ketosis, Starvation

## Abstract

**Background:**

Ketogenic diets are high fat and low carbohydrate or very low carbohydrate diets, which render high production of ketones upon consumption known as nutritional ketosis (NK). Ketosis is also produced during fasting periods, which is known as fasting ketosis (FK). Recently, the combinations of NK and FK, as well as NK alone, have been used as resources for weight loss management and treatment of epilepsy.

**Methods:**

A crossover study design was applied to 11 healthy individuals, who maintained moderately sedentary lifestyle, and consumed three types of diet randomly assigned over a three-week period. All participants completed the diets in a randomized and counterbalanced fashion. Each weekly diet protocol included three phases: Phase 1 - A mixed diet with ratio of fat: (carbohydrate + protein) by mass of 0.18 or the equivalence of 29% energy from fat from Day 1 to Day 5. Phase 2- A mixed or a high-fat diet with ratio of fat: (carbohydrate + protein) by mass of approximately 0.18, 1.63, or 3.80 on Day 6 or the equivalence of 29%, 79%, or 90% energy from fat, respectively. Phase 3 - A fasting diet with no calorie intake on Day 7. Caloric intake from diets on Day 1 to Day 6 was equal to each individual’s energy expenditure. On Day 7, ketone buildup from FK was measured.

**Results:**

A statistically significant effect of Phase 2 (Day 6) diet was found on FK of Day 7, as indicated by repeated analysis of variance (ANOVA), F(2,20) = 6.73, p < 0.0058. Using a Fisher LDS pair-wise comparison, higher significant levels of acetone buildup were found for diets with 79% fat content and 90% fat content vs. 29% fat content (with p = 0.00159**, and 0.04435**, respectively), with no significant difference between diets with 79% fat content and 90% fat content. In addition, independent of the diet, a significantly higher ketone buildup capability of subjects with higher resting energy expenditure (R^2^ = 0.92), and lower body mass index (R^2^ = 0.71) was observed during FK.

**Electronic supplementary material:**

The online version of this article (doi:10.1186/s12937-015-0028-x) contains supplementary material, which is available to authorized users.

## Background

Ketosis or ketoacidosis is a physiological state sharing an outcome of increased ketone levels in the blood due to relatively high lipid oxidation rates. Monitoring rapid and dramatic changes in ketones offers us valuable diagnoses for lipid oxidation and metabolism [1]. Several studies have clearly demonstrated that metabolic imbalance in type I diabetes has led to ***ketoacidosis (KAD)*** of blood, leading to elevated ketone levels with arterial pH < 7.3 and bicarbonate < 15 mEq/L, and causing arresting of major organ functions [2]. In addition to acidosis, studies have also shown that elevated ketone levels are a natural metabolic response to negative energy balance, wherein caloric intake is smaller than total energy expenditure, and the body burns stored fat to produce the needed energy [3], leading to a state of ketosis known as ***fasting ketosis (FK)***. FK has been used as an indicator of the effectiveness of weight loss [4-6]. Furthermore, ketosis also occurs in situations where caloric intake equals total energy expenditure, specifically in a diet that contains high percentage of fat (>60%) and/or low carbohydrate. This state of ketosis has been referred to as ***nutritional ketosis (NK)*** [7,8]. NK has been investigated as a treatment for epilepsy because ketones are thought to provide energy to the brain, which reduces epileptic seizures [9,10]. In addition, ketosis buildup capability resulting from a combination of NK and FK has been associated with weight loss efficiency and positive health outcomes [11-13].

While KAD, FK, and NK are well-defined clinical and physiological states that can produce high levels of ketones, there are other conditions, such as ***exercise*** that can actually decrease ketone levels in the blood by using ketone as an energy source in the muscles [14,15]. For the reasons described above, ketone levels are affected by several factors, such as energy balance, diet composition, and physical activities, all of which underscore the importance of determining the accuracy of ketone levels. Previous studies, including KAD, FK, NK, and exercise-related ketosis have significantly advanced the field of ketosis. However, analyses including characterization of analytical, physiological, and behavioral conditions are needed in the literature to improve the understanding of ketone level profiles in connection with monitoring of lipid oxidation, generation, utilization, and clearance of ketones under free-living conditions.

Under ketosis or ketoacidosis, the liver metabolizes fatty acids to produce two water-soluble types of ketones: acetoacetic acid and beta-hydroxybutyric acid. A third type of ketone, i.e., acetone, is also produced by the enzymatic decarboxylation of acetoacetic acid. Due to its high vapor pressure, acetone crosses the membrane barrier into the alveoli of the lung and the airway. As a result, acetone is normally present in breath. Breath acetone has been considered a reliable indicator of ketosis in adults consuming ketogenic meals [16] and can be used to predict plasma ketone bodies in children with epilepsy who are on a ketogenic diet [17]. Most recently, breath acetone has been used as a new ketone biomarker because it is non-invasive, convenient, and an accurate reflection of the body’s ketone level [18].

In the present work, our focus is on both intermittent high-fat diets (NK) and fasting diets (FK) with an aim to: 1) evaluate the effectiveness of combined NK and FK in ketone buildup capability; and 2) study how ketone buildup capability is associated with intrinsic characteristics of individuals. First, we systematically studied the effect of fat-rich diets on fasting ketone levels to determine if lipid metabolism can respond to the fat content in different diets. Diets with different fat content were provided to 11 healthy individuals in order to maintain their energy balance (i.e., calorie intake equals to energy expenditure). Breath acetone detection was then used to analyze the lipid metabolism response of these individuals during a fasting day after the high-fat diet day.

Second, we analyzed how the acetone buildup capability of the healthy individual depended on resting energy expenditure and physical characteristics, such as body mass index and body fat percentage.

In this diet study, we used Selected Ion Flow Tube-Mass Spectrometry (SIFT-MS) as a primary method for monitoring trace acetone gas in exhaled breath [19-21]. A high level of ketosis shown by elevated acetone in exhaled breath as measured by SIFT-MS was verified against measurements obtained with the commonly used blood and urine ketone detection method. Mass spectrometer-based methods, especially SIFT-MS, are currently the proven technology for most accurate detection of ketone levels in breath [16,18,22]. Blood ketone was measured using ketone strips with Precision Xtra meter, which have proven to be highly precise [23,24]. Urinary ketone was determined using Ketostix strips, as recommended by the American Diabetes Association for monitoring ketones in urine [25].

## Methods

### Subjects

Eleven (11) healthy volunteers (7 male and 4 female) with an average age of 27 ± 7 years and average BMI of 23.1 ± 5 participated in the study. Physical activity levels (PAL) of the subjects were classified based on reported frequency, duration, and type of physical activities from the Compendium of Physical Activities [26]. Additionally, all subjects completed the International Physical Activity Questionnaire (IPAQ – 7 day, self-administered format), which is widely used to classify a population’s PAL as “Low”, “Moderate”, or “High” [27]. Based on the responses, the PAL for all subjects in this study was classified as “Low” or sedentary lifestyle. The characteristics of each subject are summarized in Table 1. None of the subjects was on regular medication, nor had any history of respiratory diseases or diabetes, which enabled the comparison of ketone buildup capabilities under conditions of healthy acid-base balance and alveolar gas exchange [28]. All the subjects were educated about the study design and purpose. An informed consent form approved by the Institutional Review Board at Arizona State University (IRB protocol number: 1012005855) was obtained from each subject prior to the study.

**Table 1:** **Physiological and metabolic parameters of 11 subjects enrolled in the study**

Subject	Age	Gender	Weight (kg)	Height (m)	BMI	Body fat %	TEE (kcal/day)
1	28	M	79.1	1.68	28.0	24.2	1899
2	41	F	49.4	1.69	17.3	13.1	1398
3	35	M	77.1	1.88	21.8	10.8	1800
4	21	M	65.6	1.82	19.8	8.6	3030
5	24	M	90.5	1.68	32.1	29.0	2283
6	23	M	70.0	1.80	21.6	12.4	2592
7	37	F	63.3	1.61	24.4	33.3	2112
8	23	M	85.5	1.78	27.0	21.4	1969
9	23	M	94.3	1.93	25.3	20.0	3398
10	25	F	52.8	1.70	18.3	10.8	2232
11	20	F	52.5	1.69	18.4	12.4	2238

M: Male, F: Female, BMI: Body Mass Index, Body Fat %: represents values assessed by bio-impedance method (see text for details), and TEE: Total Energy Expenditure.

### Physiological and metabolic parameters

Total energy expenditure (TEE) for each subject of the study was estimated according to TEE = PAL * REE, where REE was the Resting Energy Expenditure assessed by indirect calorimetry method, using a mobile metabolism tracker named “Breezing” from Breezing Co., AZ (www.breezing.com); and PAL was the Physical Activity Level defined by IPAQ, and quantified as follows: PAL (female) = 1.179, PAL (male): 1.274 [29]. The sensing principle of the indirect calorimeter used for REE [30] was validated against the Douglas bag method [31]. Resting condition of the subjects for assessment of REE included overnight fasting, 12-hour period of no strenuous exercise, no-caffeine ingestion prior to the measurement, and a resting position for assessment that required the subject to hold his/her head on a comfortable surface. The subjects rested for 10 minutes before the measurement, and were instructed to relax and breathe normally during the measurement.

A bioelectrical impedance analysis using body composition analyzer SC-240 from Tanita Co. (http://www.tanita.com) was conducted to measure the percentage of body fat. In addition, weight, height, and BMI (ratio of weight-to-height squared (Kg/(meters)^2^)) were assessed for each subject.

### Diet study design

**Figure 1:**
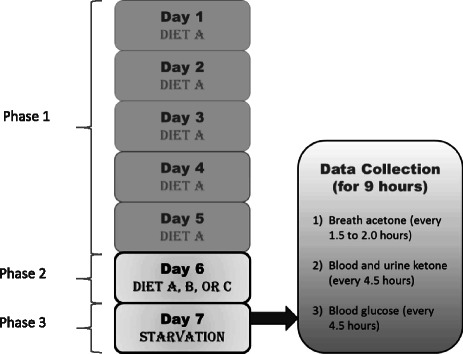
Layout of the NK and FK diet study portion of the present work.

Three different diets (Diets A, B, and C) with varying ratios of fat to total carbohydrate and protein by mass of approximately 0.18, 1.63, and 3.80 (Table 2) were applied to the study (Figure 1). In other words, it was the equivalence of 29%, 79%, and 90% of daily dietary caloric intake coming from fat for Diets A, B, and C, respectively. Diet A was high in carbohydrate and low in fat; while Diet C was the opposite. In Diet B, even though % energy from fat was lower, it was still a dominant energy source with equal contributions from carbohydrates and protein. Detailed diet composition for each subject for all three diets can be reviewed in the Additional file 1.

**Table 2:** **Example of diet compositions used in the study for subject** #**1 with total energy expenditure of 1899 kcals/day**

	Food	Amount	Total F (g)		Total C (g)	Total P (g)	Fiber (g)	Cholesterol (mg)	Total calories (kcal)	Ratio of fat: (carb + protein) by mass
			Sat. F (g)	Unsat. F (g)						
**Diet A**	Black Coffee (no cream, no sugar)	2 cup	0.0	0.0	0.0	0.6	0.0	0.0	2	**0.18**
	Classic pork breakfast sausage	1 patty	2.0	3.5	0.0	4.0	0.0	40.0	66	
	Yoplait Yogurt	1 serving	1.0	0.5	33.0	5.0	0.0	10.0	165	
	Chicken Alfredo with Fettuccine and Broccoli	1 serving	9.3	8.0	50.0	26.0	5.0	100.0	470	
	Sesame Noodle with Vegetables	1 serving	0.5	2.0	55.0	10.0	7.0	0.0	280	
	Gala Apple (small, raw, with skin)	2	0.0	0.0	32.0	0.0	5.0	0.0	132	
	Sweet and Sour Chicken with White Rice	0.5 serving	1.5	5.5	28.0	5.0	1.5	10.0	195	
	Chopped pecans	17 g	1.1	11.0	2.4	1.8	1.8	0.0	125	
	Beef Steak and Noodles	1 serving	6.0	8.0	51.0	33.0	4.0	105.00	462	
	**Total**		**60.4**		**251.4**	**85.4**	**24.3**	**265**	**1897**	
	**% Energy from food**		**29%**		**53%**	**18%**				
**Diet B**	Classic pork breakfast sausage	4 patty	8.0	14.0	0.0	16.0	0.0	160.0	262	**1.63**
	Pine nuts	57 g	5.2	63.2	22.8	11.4	3.1	0.0	752	
	Deli Delux American Cheese	2 slice	8.0	6.0	0.0	8.0	0.0	40.0	158	
	Avocado	95 g	2.0	11.3	7.6	1.9	6.5	0.0	158	
	Philadelphia Cream cheese	10 g	1.9	1.3	0.4	0.7	0.0	11.0	33	
	Roasted Almonds	84 g	3.0	42.0	15.0	18.0	9.0	0.0	537	
	**Total**		**165.9**		**45.8**	**56**	**46.9**	**211**	**1,900**	
	**% Energy from food**		**79%**		**9%**	**12%**				
**Diet C**	Mild sausage patties	6 patty	27.5	44.5	0.0	24.0	0.0	1500.0	744	**3.80**
	Avocado	64 g	1.3	7.7	5.1	1.3	4.4	0.0	106	
	Heavy whipping cream	85 mL	21.6	12.4	0.0	0.0	0.0	117.0	306	
	Chopped pecans	41 g	2.7	26.6	5.9	4.4	4.3	0.0	305	
	Smart balance buttery spread	43 g	8.3	18.2	0.0	0.0	0.0	0.0	238	
	Pine nuts	15 g	1.4	16.6	6.0	3.0	1.0	0.0	198	
	**Total**		**188.8**		**17**	**32.7**	**9.7**	**1617**	**1,897**	
	**% Energy from food**		**90%**		**3%**	**7%**				

Sat. F: saturated fat, Unsat. F: unsaturated fat.

Each subject’s physical and metabolic parameters, including height, weight, percentage of body fat, resting energy expenditure (REE), and physical activity level (PAL), were measured before the start of the study. TEE was calculated from each individual’s REE and PAL.

The study was carried out over a three-week period on each subject. Table 2 describes in detail the fat:(carbohydrate + protein) ratio by mass, as well as an example of the nutritional information of each diet. Foods with similar nutrition facts could be exchanged to satisfy personal taste as long as fat:(carbohydrate + protein) ratio by mass and TEE remained the same. The subjects followed 3 diet protocols. Each diet protocol included 3 phases with identical phases 1 and 3, and a variable phase 2 (Figure 1), as follows:

- Phase 1: The subjects followed Diet A on the first five days (Day 1-5) of the week. Diet A included a fat:(carbohydrate + protein) ratio by mass of approximately 0.18 or 29% of energy from fat.
- Phase 2: The subjects followed a randomized diet of Diet A, B, or C, with ratio of fat:(carbohydrate + protein) by mass of approximately 0.18, 1.63, or 3.80 (29%, 79%, or 90% of energy from fat), respectively on the sixth day (Day 6) of the week.
- Phase 3: The subjects followed a fasting protocol where only non-caloric drinks were allowed on the 7^th^ day (Day 7) of the week.

Each of the 11 subjects was randomly assigned among the three diet protocol over three weeks of the study, which took place from May 2013 through August 2013. Each of the 11 subjects completed all 3 diet interventions in a randomized and counterbalanced fashion. The subjects were provided with the food during Day 1-6 and ate the food under free-living conditions. On the morning of Day 7 (fasting day), the subjects were questioned to ensure diet compliance. The subjects remained at the research facility performing desk work (mostly computer work), and the research personnel who interacted with the subjects throughout the day, assessed compliance to fasting.

### Measurement protocol

The measurement protocol consisted of breath ketone (acetone) measurements performed at baseline, and during acetone buildup.

#### Baseline acetone levels

The measurements were taken after phase 1, and right before starting phase 2, in the morning of Day 6 at 10:00 am. The baseline levels reflected the acetone level after intake of Diet A on the previous days.

#### Acetone buildup levels

These measurements were taken after phase 2 at 12 hours from the last meal on Day 6 (which was consumed at 10:00 pm), and at the beginning of Phase 3 starting at 10:00 am in the morning of Day 7 (fasting day) for a period of 9 hours every 1.5 to 2 hours.

The measurement procedure was repeated during each weekly diet protocol. The acetone buildup was calculated as the difference between the acetone levels measured after 21 hours of fasting (Day 7) and the baseline level determined in the morning (before breakfast) of phase 2 (Day 6, after consumption of diet A, which was a carbohydrate-rich diet and low-fat diet on Day 5). The method of determining the baseline was chosen over a random measurement of acetone during a regular or uncontrolled day of the subject since breath acetone is known to be affected by diet and physical activity during the day [32].

### Breath ketone measurements

Concentration of acetone was assessed from exhaled breath using Selected Ion Flow Tube - Mass Spectrometer (SIFT-MS) (Instrument Science, Profile Series, Crewe, UK) in multiple ion monitoring (MIM) modes. H_3_O^+^ (m/z = 19) was chosen as the precursor ion for reaction with breath samples with ultra-high purity (99.999%) helium as the carrier gas. Precursor ion peaks at m/z of 19, 37, 55, and 73 corresponding to hydrated H_3_O^+^.nH_2_O (n = 0, 1, 2, 3) and product ion peaks after reaction with acetone at m/z of 59 and 77 corresponding to C_3_H_7_O^+^ and its hydrate C_3_H_7_O^+^.H_2_O, which were monitored. Quantification of the concentration was performed in the MIM mode by taking into account the known reaction rate coefficients for H_3_O^+^ and acetone reaction, and the measured ion flow velocity [22]. It should be noted that this method does not measure the dihydrate of protonated acetone at m/z of 95, which represents about 10-15% of total signal combining m/z of 59, 77, and 95. Individual subject breath was collected in a 6 L clean bag before immediately being measured with SIFT-MS using a pump at a constant flow rate. The flow rate was monitored and adjusted in the analysis by monitoring the water vapor level in the samples.

### Other measurements

Blood and urine ketone as well as blood glucose levels were measured every 4.5 hours for correlations with breath ketone levels. Blood ketones were measured using Precision Xtra, an electrochemical capillary blood monitor from Abbott. This monitor determined the blood ketone: beta-hydroxybutyrate (β-OHB). Standard operation procedures as prescribed by the monitor were used for the analysis. The test meter was turned on while a ketone strip was inserted to prepare for the test. The subjects’ fingertips were cleaned with an alcohol swab and dried before being pricked with the provided lancing device. A drop of blood was applied to the assigned spot of the ketone strip. Ketone levels were read from the display 10 seconds after blood was delivered to the meter.

Meanwhile, urinary ketone measurements were performed using over-the-counter reagent strips for urinalysis (Ketostix from Bayer). The strip monitored acetoacetic acid (AcAcA), upon reaction with nitroprusside salt. The reagent end of the strip was passed through the urine stream, changing the color on the strip. The color was then compared to the color chart provided with the product 15-30 seconds after the reaction.

In addition to ketone analysis, blood glucose was measured for comparison using Precision Xtra, an electrochemical capillary blood monitor from Abbott, and glucose strips, according to the standard procedure as prescribed by the vendors. All ketones and blood glucose measurements were carried out simultaneously for direct comparison.

### Statistical analysis

OriginPro software was used for all statistical analysis. The effect of a high-fat diet on the level of ketosis was analyzed for statistical significance by performing a two-way repeat measurement analysis of variance (ANOVA). Pair-wise comparisons of three data sets obtained from 11 subjects were performed using Fisher LSD tests. The data sets were related to the acetone buildup concentration due to the three different diets consumed on separate study weeks. In addition, relationships between ketosis and physiological parameters of the subjects were analyzed using nonlinear regression models keeping the physiological parameter as the independent variable.

## Results and discussions

### Effect of diet on ketosis

**Figure 2:**
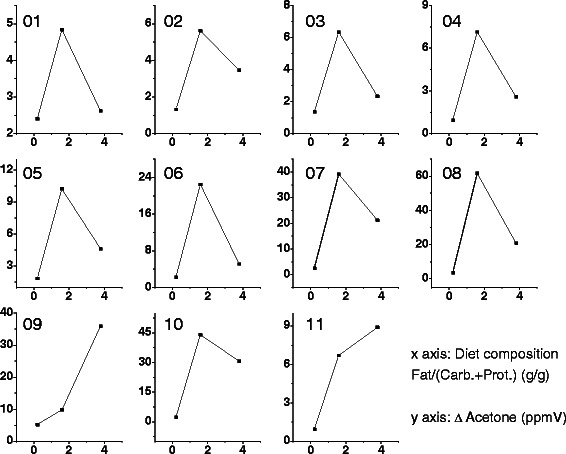
Changes in acetone during phase 3, starvation, after different diet compositions for 11 subjects. The diets are defined by the ratio of fat to total carbohydrate and protein by mass, which are plotted on the x-axis. The change in acetone in ppmV is plotted on the y-axis.

As mentioned earlier, each week included a different diet taken during phase 2 (Day 6) (Figure 1). The diets (Diets A, B, and C) differed in the ratios of fat vs. carbohydrate and protein as shown in Table 2. Figure 2 shows the acetone buildup levels as a function of the ratios of fat to total carbohydrate and protein by mass of the three diets. Table 3 summarizes the results from Figure 2, and related statistical tests, and analysis of differential increase percentage of acetone buildup concentration of one diet with respect to another (see below for more details). Figure 2 clearly shows that fasting induced significant increases in breath acetone levels after each weekly diet (Diet A, B, or C). To evaluate statistical differences between the 3 groups, analysis of variance (ANOVA) was performed, and showed F(2, 20) = 6.73, p < 0.0058, which indicated the groups are statistically different in significant ways, for a p value of 0.05.

**Table 3:** **Comparative table of the effect of Diet A, Diet B, and Diet C on acetone buildup concentration under fasting conditions**

Index	Final Acetone Buildup Concentration (ppmV)			Difference of Acetone Buildup Concentration (ppmV)		
	Diet A	Diet B	Diet C	∆ _ Diet B-Diet A _	∆ _ Diet C-Diet A _	∆ _ Diet B-Diet C _ (%)
Subject 1	2.40	4.84	2.62	2.44 (102%)	0.22 (9%)	2.22 (+85%)
Subject 2	1.32	5.63	3.46	4.30 (327%)	2.14 (162%)	2.17 (+62%)
Subject 3	1.37	6.33	2.33	4.97 (362%)	0.97 (70%)	4.00 (172%)
Subject 4	0.94	7.13	2.60	6.19 (659%)	1.66 (177%)	4.53 (174%)
Subject 5	1.85	10.24	4.63	8.39 (454%)	2.78 (150%)	5.61 (121%)
Subject 6	2.22	22.49	5.15	20.27 (913%)	2.93 (132%)	17.35 (337%)
Subject 7	2.43	39.18	21.24	36.75 (1512%)	18.81 (774%)	17.94 (84%)
Subject 8	3.73	61.76	20.83	58.03 (1556%)	17.10 (458%)	40.93 (196%)
Subject 9	5.27	9.81	35.93	4.54 (86%)	30.66 (582%)	−26.12 (−72%)
Subject 10	2.28	44.04	30.75	41.76 (1832%)	28.47 (1249%)	13.29 (43%)
Subject 11	0.94	6.71	8.89	5.77 (614%)	7.95 (846%)	−2.18 (−24%)
Mean	2.25	19.83	12.58	100% data show final acetone buildup concentration from Diet B > acetone buildup concentration from Diet A	100% data show final acetone buildup concentration from Diet C > acetone buildup concentration from Diet A	82% data show final acetone buildup concentration from Diet B > acetone buildup concentration from Diet C
Standard Deviation	1.29	19.65	12.40			
ANOVA p value	<0.0058					
Fisher Test p value of _Diet A and Diet B_	0.00159**					
Fisher Test p value of _Diet A and Diet C_	0.04435**					
Fisher Test p value of _Diet B and Diet C_	0.14797*					

∆_Diet B-Diet A:_ Difference of Acetone Buildup Concentration between Diet B and Diet A.

∆_Diet C-Diet A:_ Difference of Acetone Buildup Concentration between Diet C and Diet A.

∆_Diet B-Diet C_: Difference of Acetone Buildup Concentration between Diet B and Diet C.

“%” indicates the percentage difference between acetone buildup concentration of:

1) Diet B with respect to Diet A, taking Diet A as reference: [(Diet B – Diet A) / Diet A] x 100;

2) Diet C with respect to Diet A, taking Diet A as reference: [(Diet C – Diet A) / Diet A] x 100;

3) Diet B with respect to Diet C, taking Diet C as reference: [(Diet C – Diet B) / Diet C] x 100.

“**” indicates: Significant difference for p = 0.05, “*” indicates: No significant difference for p = 0.05.

In order to further compare the effect of the different diets on the acetone buildup concentration, a Fisher LDS pair-wise comparison was performed (Table 3). For p value of 0.05, the results indicated significant difference between Diet B with respect to Diet A (p = 0.00156**), and Diet C with respect to Diet A (p = 0.04435**), but no significant difference between Diet B with respect to Diet C (p = 0.14797*). It is noteworthy that 1) higher fat ratio diets (Diet B, and Diet C) produced statistically significant acetone buildup concentrations than the lower fat ratio diet (Diet A), 2) higher fat ratio diets (Diet B and Diet C) did not show statistically significant differences in the acetone buildup concentrations, meaning that levels of acetone buildup produced from a 79% fat content diet (Diet B = 1.63:1 fat:(carbohydrates + protein)) were statistically similar to those levels produced from a 90% fat content diet (Diet C = 3.80:1 fat:(carbohydrates + protein)).

Furthermore, analysis of differential increase percentage of acetone buildup concentration at individual levels (Table 3) indicated the following:

- *Diet B vs. Diet A:* 100% of individuals showed increase in acetone buildup concentration above 86% and up to 1800% when comparing Diet B with Diet A, using Diet A as reference (This is in line with Fisher LDS test).
- *Diet C vs. Diet A:* 100% of the individuals showed increase in acetone buildup concentration above 9% and up to 1248% when comparing Diet C with Diet A, using Diet A as reference (This is in line with Fisher LDS test).
- *Diet B vs. Diet C:* 82% of the individuals showed increase in acetone buildup concentration above 43% and up to 337% when comparing Diet B with Diet C, using Diet C as reference.

With regard to the last comparison (*Diet B vs. Diet C*), it is noteworthy that Diet B and Diet C did not show statistically significant differences in acetone buildup concentrations at population levels due to intrinsically high dispersion of the values across the population (mean ± SD of Diet B = (19.83 ± 19.64), mean ± SD of Diet C = (12.58 ± 12.40) ppmV). However, an analysis of the differences between acetone buildup concentrations at individual levels indicates 9 of 11 individuals had higher acetone buildup with the relatively lower fat ratio diet (Diet B = 79% fat content, 1.63:1 fat:(carbohydrates + protein)) compared to the higher fat diet (90%, 3.80:1 fat:(carbohydrates + protein)). Therefore, the higher fat diet level did not show higher buildup to higher levels of ketosis, and some individuals may have a better development of ketosis with lower 79% vs 90% fat content diet.

The plateau in ketosis at higher fat content diet (Diet C) suggest two potential implications: 1) Excessive ketone production may induce more ketone utilization as a fuel for the heart, muscle and/or brain [3]; 2) Ketone production may shut down when excessive ketone levels are produced. Since, the ketone level in breath is reflective of ketone level in blood, and these levels are a consequence of production vs. utilization, the cause of the most dominant factor responsible for Diet C’s outcome remains an open question. However, it is worthy to note that a *reduction of fat in diet to meet a ratio of 1.63:1 fat to carbohydrate and protein ratio (Diet B) may lead to a ketosis state that is at least as efficient as the ketosis buildup at high fat diet content of 3.80:1 fat:(carbohydrates + protein)) (Diet C). This eliminate cumbersome ultra-high fat needs in ketogenic diets*, which are typically associated with nausea and non-compliance [33,34]. A reduction in fat ratio also would mean more food choices to be available which may improve the diet compliance especially in epileptic children under such diets.

**Figure 3:**
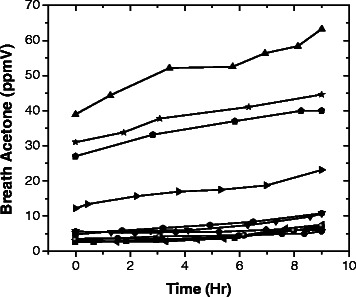
Breath ketone, acetone, measured during the starvation day (Day 6) for 11 subjects after Diet B. Time zero was set for 10:00 am in the morning, after 12 hours from last meal (10:00 pm on Day 5).

In addition, there are several significant observations in the kinetics of acetone buildup among individuals. As an example, Figure 3 shows the acetone buildup among the 11 subjects during starvation (phase 3: Day 7) following Diet B (1.6:1 fat:(carbohydrates + protein)) on Day 6 (phase 2). The data shows that individuals are different not only in their acetone levels but also in their acetone growth kinetics. This variability indicates the need for personal monitoring of breath acetone in order to choose the most effective diets for development of ketosis in healthy individuals, which leads to investigation of the physical and physiological factors in individuals that determine acetone buildup capability.

**Figure 4:**
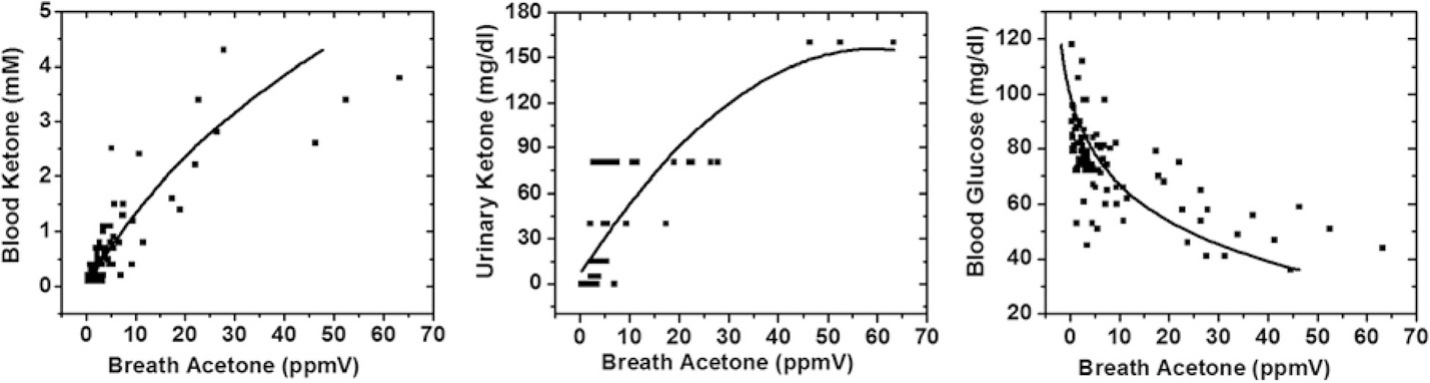
Correlation of breath acetone levels with blood ketone and urine ketone as well as blood glucose collected from different subjects on their fasting days.

Figure 4 shows correlations of breath acetone levels with blood ketone, urinary ketone, and blood glucose. The very high breath acetone levels collected from all 11 subjects were confirmed with the high blood ketone and urinary ketone levels. In addition, the exponential decay relationship between breath acetone and blood glucose also indicated that blood glucose was depleted as breath acetone was produced.

### Ketone buildup capability

**Figure 5:**
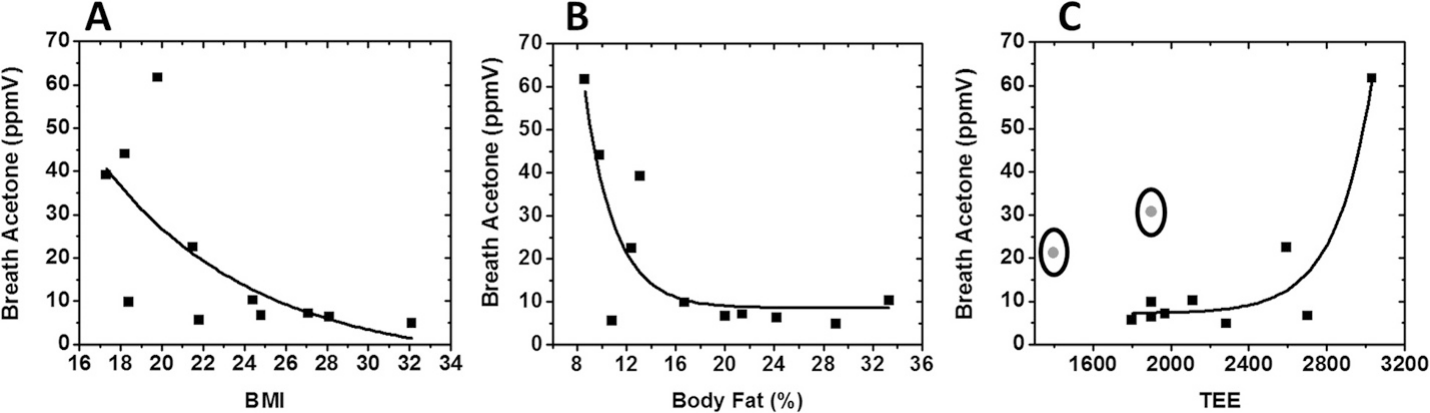
Correlation of acetone buildup on starvation day (Day 6) after diet C (on Day 5) with different physiological parameters. **(A)** Exponentially decaying correlation of acetone buildup with BMI (R^2^ = 0.46), **(B)** Exponentially decaying correlation of acetone buildup with Body Fat % (R^2^ = 0.71), and **(C)** Exponentially increasing correlation of acetone buildup with TEE (TEE = PAL × REE, for more details see experimental section) (R^2^ = 0.92 for n = 9). Two subjects with lowest BMI (marked in circles) did not follow the trend likely due to non-compliance of energy balanced diet on Day 5 of the diet.

The increase in acetone levels so-called *fasting ketosis capability* was correlated with different physiological and physical parameters recorded during the study. Figure 5 shows parameter relationships and correlation plots for acetone buildup for Day 7 of Diet C with BMI, TEE, and body fat percentage (%). There was an exponential decay tendency in the acetone production with both BMI (R^2^ = 0.46) and body fat % (R^2^ = 0.71). Though the correlation was weaker with BMI, both correlations still presented the general tendency that subjects with lower BMI produced more acetone. This finding was in agreement with former assessments presented in literatures [35,36]. At the beginning of the day, 12 hours after the last meal, collected breath acetone in each individual showed expected levels. All subjects on Diet A of low fat/high carbohydrates, produced less than 1ppmV acetone; while subjects on Diet B and C, ketogenic diets, produced significantly higher acetone levels.

The increase in breath acetone levels has previously been reported by Smith, Španěl et al. [32,37]. However, the absolute values of acetone buildups from this study are higher than previously reported studies (as seen in Table 4). The reasons of the differences are rationalized as follows:

**Table 4:** **Comparative table of acetone buildup conditions for this study and other studies**

Reference	Conditioning Diet		Diet prior to acetone testing		Condition during testing	
	Period	Composition	Period	Composition	Condition	Period
This work	Day 1-5	fat:(carbohydrate + protein) by mass of 0.18 or the equivalence of 29% energy from fat	Day 6	Diet B*: fat:(carbohydrate + protein) by mass of 1.63, or 78% energy from fat	Fasting since last meal	22 hrs
Ref. 37			Single meal	liquid protein-energy meal**: Fat:carbohydrate + protein) by mass: 12 g/49 g; 0.24	Fasting since last meal	4-5 hrs
Ref. 32					Diet with 20 g carbohydrate/day***	8 hrs/day during 12 days

Note: * highest diet for acetone build, ** liquid protein-energy meal: Total Fat: 12 g, Total Carbs: 31 g, Protein: 18 g; Fat: (carbohydrate + protein) by mass = 12 g/49 g = 0.24; *** No information on content of fat or proteins is provided. In addition, no level of energy balance (negative or equilibrated) is provided to determine whether the subjects had any additional effect from calorie restriction.

1. We have observed that during a fasting period, the time from last meal triggers an exponential growth in acetone levels. Our fasting times are considerably longer, 2.5 to 3 times longer than previously reported values from literature, which were 8 hours at the most (e.g. from 8:00 am to 4:00 pm) [32].
2. Furthermore, the fat content of the diets administered 22 hours prior to the fasting period of this study are at least 3 to 4 times higher than those used in the previously reported ketogenic diet studies [37].

As a result, this study shows higher levels compared to the previous studies due to higher fat content in the diets combined with a longer fasting period of 22 hours.

The higher levels of ketone buildup were also verified by high urine and blood ketone measurements (up to 4.4 mM), as shown in Figure 4. The methods used for this verification were validated in a previous study that had successfully used the methods to screen ketosis cases in a subject’s population [38]. In addition, the assessed values of urine, blood, and breath ketone for this study were also found to correlate with the urine, blood, and breath acetone levels found in a high ketoacidosis group reported by Qiao, Hu, et al. [18].

Another interesting observation of this study is related to acetone buildup in correlation with BMI, body fat %, and REE. Subjects with lower BMI and body fat % seemed to produce higher levels of breath acetone compared to the rest. Additionally, when combining ketogenic diets with fasting for the next 9 hours, we found exponential growth of acetone levels from all subjects. Acetone levels as high as 35-65 ppmV were observed for subjects with lower BMI and body fat %.

Furthermore, the trend of acetone buildup with TEE was interesting because for nine of eleven subjects (see below), the data showed a strong exponentially increasing trend (R^2^ = 0.92). Since TEE values are a direct measure of REE values (TEE = PAL * REE, with PAL corresponding to sedentary level for all subjects of the study), the trend was indicative of the direct correlation between ketosis buildup or fasting ketosis capability and metabolic rates. This is a conclusion that requires further investigation, but in principle, it should be expected that higher metabolic rates might correlate with higher fat burning capabilities, and therefore higher ketone buildup in healthy individuals under NK and FK states. It is worth noticing, the remaining 2 subjects (outliers of the general trend), had relatively low metabolism, and with the least BMI among all the 11 subjects. An interview with the subjects the next morning revealed they did not comply with the assignment of consuming completely their total supply of food on Day 6. Therefore, the higher acetone changes may have been a result of negative energy balance (calorie intake was less than their TEE), a condition that deviates from the one applied to the other 9 subjects, and increases fat oxidation rates from the body’s stored fat.

The collected and analyzed results brought some important insights into the common high fat/low carbohydrate or very low carbohydrate ketogenic diets, particularly as they are applied to individuals. Higher fat content diet did not always induce the most breath acetone, which correlates to fat oxidation. However, more in-depth research needs to be performed to draw further conclusions since our study only examined ketosis buildup over one day of a ketogenic diet and one day of fasting. Additionally, one small drawback was that the highest fat diet was very difficult to consume. As a result, it was hard to recruit more subjects.

## Conclusions

In conclusion, the data presented in this study show that while Nutritional Ketosis (NK) is an effective way to control ketone levels, NK has a great impact on FK in terms of ketone production, which is desirable in certain cases such as seizure control in epilepsy. Though ratio of fat:(carbohydrate + protein) by mass of 3.80 or the equivalence of 90% energy from fat, a high-fat diet that is most commonly recommended in NK for treatments, the data from this paper show that it may not be necessary for everyone. On the contrary, most subjects in this study showed similar or better ketosis buildup with a diet of lower fat content. These observations suggest the need for personalized monitoring of individuals for optimization of their diet. Variations in the subject’s ability to produce acetone were found to correlate with several physiological and physical parameters. For example, the correlations indicated that subjects with lower fat percentage, BMI, as well as higher metabolic rate (REE) have higher ketone buildups, and thus metabolize fat more efficiently.

## Additional file

Additional file 1:
**Supplementary data.**
