# Supplementary material for: Acetone as biomarker for ketosis buildup capability - a study in healthy individuals under combined high fat and starvation diets
Source: Nutr J. 2015 Apr 22;14:41. doi: 10.1186/s12937-015-0028-x (PMC4471925; doi:10.1186/s12937-015-0028-x)
Supplement: Additional file 1: — Supplementary data. [file 12937_2015_28_MOESM1_ESM.docx]

**Supplementary section**

**Acetone as biomarker for ketosis buildup capability - a study in healthy individuals under combined high fat and starvation diets**

Amlendu Prabhakar*^1^, Ashley Quach*^1^, Haojiong Zhang^1^, Mirna Terrera^1^, David Jackemeyer^1^, Xiaojun Xian^1^, Francis Tsow^1^, Nongjian Tao^1,3^, and Erica S. Forzani^1,2^**

^1^Center for Bioelectronics and Biosensors, the Biodesign Institute, Arizona State University. ^2^School for Engineering of Matter, Transport, and Energy, Arizona State University.

^3^School of Electrical, Computer, and Energy Engineering, Arizona State University.

This section shows a detail of Diet A, B, and C consumed by all the subjects of the study. The type of food, amount, total fat (F), carbohydrate (C), protein (P), fiber, and cholesterol mass (g), total calories (kCal), and the ratio of fat vs. carbohydrate and protein are specified together with the percentage (%) of energy from fat, carbohydrate and protein.

**Table S1: Detailed diet compositions used in the study for subject #1.**

|  | **Food** | **Amount** | **Total F (g)** | | **Total C (g)** | **Total P (g)** | **Fiber (g)** | **Cholesterol (mg)** | **Total calories (kcal)** | **Ratio of fat: (carb+protein) by mass** |
| --- | --- | --- | --- | --- | --- | --- | --- | --- | --- | --- |
|  |  |  | **Sat. F (g)** | **Unsat. F (g)** |  |  |  |  |  |  |
| **Diet A** | Black Coffee (no cream, no sugar) | 2 cups | 0.0 | 0.0 | 0.0 | 0.6 | 0.0 | 0.0 | 2 | **0.18** |
|  | Classic pork breakfast sausage | 1 patty | 2.0 | 3.5 | 0.0 | 4.0 | 0.0 | 40.0 | 66 |  |
|  | Yoplait Yogurt | 1 serving | 1.0 | 0.5 | 33.0 | 5.0 | 0.0 | 10.0 | 165 |  |
|  | Chicken Alfredo with Fettuccine and Broccoli | 1 serving | 9.3 | 8.0 | 50.0 | 26.0 | 5.0 | 100.0 | 470 |  |
|  | Sesame Noodle with Vegetables | 1 serving | 0.5 | 2.0 | 55.0 | 10.0 | 7.0 | 0.0 | 280 |  |
|  | Gala Apple (small, raw, with skin) | 2 | 0.0 | 0.0 | 32.0 | 0.0 | 5.0 | 0.0 | 132 |  |
|  | Sweet and Sour Chicken with White Rice | 0.5 serving | 1.5 | 5.5 | 28.0 | 5.0 | 1.5 | 10.0 | 195 |  |
|  | Chopped pecans | 17g | 1.1 | 11.0 | 2.4 | 1.8 | 1.8 | 0.0 | 125 |  |
|  | Beef Steak and Noodles | 1 serving | 6.0 | 8.0 | 51.0 | 33.0 | 4.0 | 105.00 | 462 |  |
|  | **Total** |  | **60.4** | | **251.4** | **85.4** | **24.3** | **265** | **1897** |  |
|  | **% Energy from food** |  | **29%** | | **53%** | **18%** |  |  |  |  |
|  | | | | | | | | | | |
| **Diet B** | Classic pork breakfast sausage | 4 patty | 8.0 | 14.0 | 0.0 | 16.0 | 0.0 | 160.0 | 262 | **1.63** |
|  | Pine nuts | 57g | 5.2 | 63.2 | 22.8 | 11.4 | 3.1 | 0.0 | 752 |  |
|  | Deli Delux American Cheese | 2 slice | 8.0 | 6.0 | 0.0 | 8.0 | 0.0 | 40.0 | 158 |  |
|  | Avocado | 95g | 2.0 | 11.3 | 7.6 | 1.9 | 6.5 | 0.0 | 158 |  |
|  | Philadelphia Cream cheese | 10g | 1.9 | 1.3 | 0.4 | 0.7 | 0.0 | 11.0 | 33 |  |
|  | Roasted Almonds | 84g | 3.0 | 42.0 | 15.0 | 18.0 | 9.0 | 0.0 | 537 |  |
|  | **Total** |  | **165.9** | | **45.8** | **56** | **46.9** | **211** | **1,900** |  |
|  | **% Energy from food** |  | **79%** | | **9%** | **12%** |  |  |  |  |
|  | | | | | | | | | | |
| **Diet C** | Mild sausage patties | 6 patty | 27.5 | 44.5 | 0.0 | 24.0 | 0.0 | 1500.0 | 744 | **3.80** |
|  | Avocado | 64g | 1.3 | 7.7 | 5.1 | 1.3 | 4.4 | 0.0 | 106 |  |
|  | Heavy whipping cream | 85mL | 21.6 | 12.4 | 0.0 | 0.0 | 0.0 | 117.0 | 306 |  |
|  | Chopped pecans | 41g | 2.7 | 26.6 | 5.9 | 4.4 | 4.3 | 0.0 | 305 |  |
|  | Smart balance buttery spread | 43g | 8.3 | 18.2 | 0.0 | 0.0 | 0.0 | 0.0 | 238 |  |
|  | Pine nuts | 15g | 1.4 | 16.6 | 6.0 | 3.0 | 1.0 | 0.0 | 198 |  |
|  | **Total** |  | **188.8** | | **17** | **32.7** | **9.7** | **1617** | **1,897** |  |
|  | **% Energy from food** |  | **90%** | | **3%** | **7%** |  |  |  |  |

**Table S2: Detailed diet compositions used in the study for subject #2.**

|  | **Food** | **Amount** | **Total F (g)** | | **Total C (g)** | **Total P (g)** | **Fiber (g)** | **Cholesterol (mg)** | **Total calories (kcal)** | **Ratio of fat: (carb+protein) by mass** |
| --- | --- | --- | --- | --- | --- | --- | --- | --- | --- | --- |
|  |  |  | **Sat. F (g)** | **Unsat. F (g)** |  |  |  |  |  |  |
| **Diet A** | Black coffee | 1 cup | 0.0 | 0.0 | 0.0 | 0.0 | 0.0 | 0.0 | 2 | **0.16** |
|  | Milk | 1/6 cup | 0.0 | 0.6 | 1.5 | 1.0 | 0.0 | 2.5 | 16 |  |
|  | Nature valley | 1 package | 0.5 | 6.0 | 29.0 | 4.0 | 2.0 | 0.0 | 190 |  |
|  | Cereal | 1 cup | 0.0 | 3.0 | 31.0 | 3.0 | 4.0 | 0.0 | 163 |  |
|  | Yogurt | 6 oz | 0.0 | 0.0 | 16.0 | 5.0 | 0.0 | 0.0 | 84 |  |
|  | Salad | 1 plate | 0.0 | 2.0 | 37.0 | 10.0 | 15.0 | 0.0 | 206 |  |
|  | Cheese | pieces | 10.0 | 10.0 | 0.0 | 14.0 | 0.0 | 60 | 236 |  |
|  | Fruit salad | 2 serving | 0.0 | 4.0 | 60.0 | 8.0 | 8.0 | 0.0 | 308 |  |
|  | **Total** |  | **36.1** | | **174.5** | **45.0** | **29.0** | **62.5** | **1205** |  |
|  | **% Energy from food** |  | **27%** | | **58%** | **15%** |  |  |  |  |
|  | | | | | | | | | | |
| **Diet B** | Avocado | 150g | 3.0 | 17.9 | 12.0 | 3.0 | 10.3 | 0.0 | 249 | **1.69** |
|  | Heavy whipping cream | 50mL | 12.5 | 7.5 | 0.0 | 0.0 | 0.0 | 75.0 | 180 |  |
|  | Roasted Almonds | 120g | 4.3 | 60.0 | 21.4 | 25.7 | 12.9 | 0.0 | 767 |  |
|  | Coffee | 2 cups | 0.0 | 0.0 | 0.0 | 0.0 | 0.0 | 0.0 | 5 |  |
|  | **Total** |  | **105.2** | | **33.4** | **28.7** | **23.2** | **75.0** | **1201** |  |
|  | **% Energy from food** |  | **79%** | | **11%** | **10%** |  |  |  |  |
|  | | | | | | | | | | |
| **Diet C** | Smart balance buttery spread | 25g | 4.8 | 10.6 | 0.0 | 0.0 | 0.0 | 0.0 | 138 | **3.80** |
|  | Avocado | 30g | 0.6 | 3.6 | 2.4 | 0.6 | 2.1 | 0.0 | 50 |  |
|  | Heavy whipping cream | 100mL | 25.0 | 15.0 | 0.0 | 0.0 | 0.0 | 150 | 360 |  |
|  | Chopped pecans | 44g | 2.9 | 28.7 | 6.3 | 4.7 | 4.3 | 0.0 | 327 |  |
|  | Pine nuts | 35g | 3.3 | 38.7 | 14.0 | 7.0 | 2.3 | 0.0 | 462 |  |
|  | Black coffee | 1 cup | 0.0 | 0.0 | 0.0 | 0.0 | 0.0 | 0.0 | 2 |  |
|  | **Total** |  | **133.2** | | **22.7** | **12.3** | **8.7** | **150.0** | **1339** |  |
|  | **% Energy from food** |  | **90%** | | **7%** | **3%** |  |  |  |  |

**Table S3: Detailed diet compositions used in the study for subject #3.**

|  | **Food** | **Amount** | **Total F (g)** | | **Total C (g)** | **Total P (g)** | **Fiber (g)** | **Cholesterol (mg)** | **Total calories (kcal)** | **Ratio of fat: (carb+protein) by mass** |
| --- | --- | --- | --- | --- | --- | --- | --- | --- | --- | --- |
|  |  |  | **Sat. F (g)** | **Unsat. F (g)** |  |  |  |  |  |  |
| **Diet A** | Cocoa 85% | 70g | 11.0 | 19.0 | 20.0 | 6.0 | 8.0 | 0.0 | 375 | **0.24** |
|  | Coffee | 3 cups | 0.0 | 0.0 | 0.0 | 0.0 | 0.0 | 0.0 | 7 |  |
|  | Trail Mix | 4 oz | 4.0 | 2.0 | 84.0 | 12.0 | 12.0 | 0.0 | 438 |  |
|  | Low Fat Yogurt | 225 g | 1.0 | 2.0 | 16.0 | 11.0 | 0.0 | 170.0 | 135 |  |
|  | Eggs | 5 | 10.0 | 15.0 | 0.0 | 30.0 | 0.0 | 350.0 | 345 |  |
|  | Salad | 1 plate | 0.0 | 2.0 | 37.0 | 10.0 | 15.0 | 0.0 | 206 |  |
|  | Fruits salad | 2 serving | 0.0 | 4.0 | 60.0 | 8.0 | 8.0 | 0.0 | 308 |  |
|  | **Total** |  | **70.0** | | **217.0** | **77.0** | **55.0** | **520.0** | **1814** |  |
|  | **% Energy from food** |  | **35%** | | **48%** | **17%** |  |  |  |  |
|  | | | | | | | | | | |
| **Diet B** | Avocado | 260g | 5.4 | 31.0 | 20.8 | 5.2 | 17.8 | 0.0 | 432 | **1.63** |
|  | Pine nuts | 10g | 1.1 | 10.9 | 4.0 | 2.0 | 1.0 | 0.0 | 132 |  |
|  | Roasted Almonds | 155g | 5.5 | 77.5 | 27.7 | 33.2 | 16.6 | 0.0 | 991 |  |
|  | Heavy whipping cream | 50mL | 12.5 | 7.5 | 0.0 | 0.0 | 0.0 | 75.0 | 180 |  |
|  | Coffee | 3 cups | 0.0 | 0.0 | 0.0 | 0.0 | 0.0 | 0.0 | 7 |  |
|  | **Total** |  | **151.4** | | **52.5** | **40.4** | **35.4** | **75.0** | **1742** |  |
|  | **% Energy from food** |  | **79%** | | **12%** | **9%** |  |  |  |  |
|  | | | | | | | | | | |
| **Diet C** | Mild sausage patties | 2 patty | 9.2 | 14.8 | 0.0 | 8.0 | 0.0 | 500.0 | 248 | **3.80** |
|  | Heavy whipping cream | 126mL | 31.5 | 18.9 | 0.0 | 0.0 | 0.0 | 225 | 454 |  |
|  | Chopped pecans | 65g | 4.0 | 42.4 | 9.3 | 7.0 | 4.2 | 0.0 | 483 |  |
|  | Pine nuts | 35g | 3.3 | 38.7 | 14.0 | 7.0 | 2.3 | 0.0 | 462 |  |
|  | Smart balance buttery spread | 15g | 2.9 | 6.3 | 0.0 | 0.0 | 0.0 | 0.0 | 83 |  |
|  | Coffee | 3 cups | 0.0 | 0.0 | 0.0 | 0.0 | 0.0 | 0.0 | 7 |  |
|  | **Total** |  | **172.0** | | **23.3** | **22.0** | **6.5** | **725.0** | **1737** |  |
|  | **% Energy from food** |  | **90%** | | **5%** | **5%** |  |  |  |  |

**Table S4: Detailed diet compositions used in the study for subject #4.**

|  | **Food** | **Amount** | **Total F (g)** | | **Total C (g)** | **Total P (g)** | **Fiber (g)** | **Cholesterol (mg)** | **Total calories (kcal)** | **Ratio of fat: (carb+protein) by mass** |
| --- | --- | --- | --- | --- | --- | --- | --- | --- | --- | --- |
|  |  |  | **Sat. F (g)** | **Unsat. F (g)** |  |  |  |  |  |  |
| **Diet A** | Coffee with cream + sugar | 4 cups | 2.0 | 3.0 | 40.0 | 5.0 | 0.0 | 10.0 | 226 | **0.16** |
|  | Beef with Broccoli and Rice | 1 serving | 1.0 | 3.0 | 25.0 | 9.0 | 2.0 | 15.0 | 170 |  |
|  | Chicken Alfredo with Fettuccine | 1 serving | 9.0 | 8.5 | 49.0 | 29.0 | 5.0 | 100.0 | 470 |  |
|  | Chicken Parmigiana | 1 serving | 7.0 | 20.0 | 47.0 | 25.0 | 7.0 | 40.0 | 530 |  |
|  | Chicken Fried Rice | 2.5 serving | 3.0 | 18.0 | 102.5 | 22.5 | 5.0 | 37.5 | 700 |  |
|  | Fettuccine Alfredo with Chicken Broccoli | 1 serving | 5.0 | 4.0 | 36.0 | 14.0 | 2.0 | 40.0 | 280 |  |
|  | Yoplait yogurt | 12 oz. | 2.0 | 1.0 | 66.0 | 10.0 | 0.0 | 20.0 | 340 |  |
|  | Banana | 2 | 0.2 | 0.6 | 54.0 | 2.6 | 6.2 | 0.0 | 233 |  |
|  | **Total** |  | **87.3** | | **419.5** | **117.1** | **27.2** | **262.5** | **2949** |  |
|  | **% Energy from food** |  | **27%** | | **57%** | **16%** |  |  |  |  |
|  | | | | | | | | | | |
| **Diet B** | Avocado | 260g | 5.4 | 31.0 | 20.8 | 5.2 | 17.8 | 0.0 | 432 | **1.63** |
|  | Heavy whipping cream | 50mL | 12.5 | 7.5 | 0.0 | 0.0 | 0.0 | 75.0 | 180 |  |
|  | Roasted Almonds | 250g | 8.9 | 125.0 | 44.6 | 53.6 | 26.8 | 0.0 | 1598 |  |
|  | Shelled walnuts | 72g | 5.1 | 41.2 | 10.3 | 10.3 | 6.8 | 0.0 | 499 |  |
|  | Black Coffee | 2 cups | 0.0 | 0.0 | 0.0 | 0.0 | 0.0 | 0.0 | 4 |  |
|  | **Total** |  | **236.6** | | **75.7** | **69.1** | **51.4** | **75.0** | **2713** |  |
|  | **% Energy from food** |  | **79%** | | **11%** | **10%** |  |  |  |  |
|  | | | | | | | | | | |
| **Diet C** | Mild sausage patties | 6 patty | 27.5 | 44.5 | 0.0 | 24.0 | 0.0 | 1500.0 | 744 | **3.80** |
|  | Heavy whipping cream | 120mL | 30.0 | 18.0 | 0.0 | 0.0 | 0.0 | 180.0 | 432 |  |
|  | Chopped pecans | 69g | 4.3 | 45.0 | 9.9 | 7.4 | 6.8 | 0.0 | 513 |  |
|  | Pine nuts | 46g | 4.6 | 50.6 | 18.4 | 9.2 | 3.4 | 0.0 | 607 |  |
|  | Smart balance buttery spread | 60g | 8.3 | 28.7 | 0.0 | 0.0 | 0.0 | 0.0 | 332 |  |
|  | Black Coffee | 2 cups | 0.0 | 0.0 | 0.0 | 0.0 | 0.0 | 0.0 | 4 |  |
|  | **Total** |  | **261.5** | | **28.3** | **40.6** | **10.2** | **1680** | **2632** |  |
|  | **% Energy from food** |  | **90%** | | **4%** | **6%** |  |  |  |  |

**Table S5: Detailed diet compositions used in the study for subject #5.**

|  | **Food** | **Amount** | **Total F (g)** | | **Total C (g)** | **Total P (g)** | **Fiber (g)** | **Cholesterol (mg)** | **Total calories (kcal)** | **Ratio of fat: (carb+protein) by mass** |
| --- | --- | --- | --- | --- | --- | --- | --- | --- | --- | --- |
|  |  |  | **Sat. F (g)** | **Unsat. F (g)** |  |  |  |  |  |  |
| **Diet A** | Low fat milk | 16oz. | 4.0 | 0.0 | 26.0 | 16.0 | 0.0 | 24.0 | 204 | **0.14** |
|  | Sesame noodles with vegetables | 1 serving | 0.5 | 2.0 | 55.0 | 10.0 | 7.0 | 0.0 | 280 |  |
|  | Roasted Almonds | 20g | 0.7 | 10.0 | 3.6 | 4.4 | 2.0 | 0.0 | 128 |  |
|  | Lean Cuisine stir fry vegetables and shrimp | 1 serving | 0.5 | 2.0 | 42.0 | 12.0 | 5.0 | 50.0 | 240 |  |
|  | Chicken fried rice | 2 cups | 4.0 | 17.0 | 70.0 | 21.0 | 2.2 | 180.0 | 550 |  |
|  | Chicken Teriyaki | 1 serving | 1.0 | 2.5 | 44.0 | 17.0 | 3.0 | 35.0 | 280 |  |
|  | Gala Apples (small) | 1 | 0.0 | 0.0 | 17.0 | 0.0 | 2.5 | 0.0 | 68 |  |
|  | Mini Truffle cheesecake | ½ serving | 5.5 | 5.0 | 15.0 | 3.0 | 1.5 | 0.0 | 175 |  |
|  | Yoplait yogurt | 6 oz. | 1.0 | 0.5 | 33.0 | 5.0 | 0.0 | 10.0 | 170 |  |
|  | **Total** |  | **56.2** | | **305.6** | **88.4** | **23.2** | **299.0** | **2095** |  |
|  | **% Energy from food** |  | **24%** | | **59%** | **17%** |  |  |  |  |
|  | | | | | | | | | | |
| **Diet B** | Avocado | 150g | 3.0 | 17.9 | 12.0 | 3.0 | 10.3 | 0.0 | 249 | **1.63** |
|  | Pine nuts | 70g | 7.4 | 76.7 | 28.0 | 14.0 | 4.1 | 0.0 | 924 |  |
|  | Roasted Almonds | 120g | 4.3 | 60.0 | 21.4 | 25.7 | 12.9 | 0.0 | 767 |  |
|  | **Total** |  | **169.3** | | **61.4** | **42.7** | **27.3** | **0.0** | **1940** |  |
|  | **% Energy from food** |  | **79%** | | **13%** | **8%** |  |  |  |  |
|  | | | | | | | | | | |
| **Diet C** | Mild sausage patties | 4 patty | 18.3 | 29.7 | 0.0 | 16.0 | 0.0 | 1000.0 | 496 | **3.80** |
|  | Dean’s guacamole dip | 82g | 6.7 | 17.8 | 5.5 | 2.7 | 0.0 | 0.0 | 254 |  |
|  | Heavy whipping cream | 140mL | 35.0 | 21.0 | 0.0 | 0.0 | 0.0 | 210.0 | 504 |  |
|  | Chopped pecans | 40g | 2.6 | 26.1 | 5.7 | 4.3 | 3.9 | 0.0 | 297 |  |
|  | Pine nuts | 23g | 2.5 | 27.5 | 10.0 | 5.0 | 1.8 | 0.0 | 330 |  |
|  | Black coffee | 1 cup | 0.0 | 0.0 | 0.0 | 0.0 | 0.0 | 0.0 | 2 |  |
|  | **Total** |  | **187.2** | | **21.2** | **28.0** | **5.7** | **1210** | **1883** |  |
|  | **% Energy from food** |  | **89%** | | **5%** | **6%** |  |  |  |  |

**Table S6: Detailed diet compositions used in the study for subject #6.**

|  | **Food** | **Amount** | **Total F (g)** | | **Total C (g)** | **Total P (g)** | **Fiber (g)** | **Cholesterol (mg)** | **Total calories (kcal)** | **Ratio of fat: (carb+protein) by mass** |
| --- | --- | --- | --- | --- | --- | --- | --- | --- | --- | --- |
|  |  |  | **Sat. F (g)** | **Unsat. F (g)** |  |  |  |  |  |  |
| **Diet A** | Black coffee + sugar + cream | 4 cups | 2.0 | 3.0 | 40.0 | 5.0 | 0.0 | 10.0 | 225 | **0.12** |
|  | Lean Cuisine Pizza | 1 serving | 2.0 | 4.0 | 57.0 | 19.0 | 3.0 | 10.0 | 358 |  |
|  | Mattar Paneer (Amy's Indian) | 1 serving | 4.0 | 7.0 | 54.0 | 13.0 | 6.0 | 20.0 | 370 |  |
|  | Vegetable Korma (Amy’s Indian) | 1 serving | 3.5 | 8.5 | 41.0 | 9.0 | 7.0 | 0.0 | 310 |  |
|  | Amy’s Soft Taco | 1 serving | 1.5 | 3.0 | 40.0 | 7.0 | 6.0 | 5.0 | 229 |  |
|  | Kidfresh Muy Cheesy Quesadillas | 1 serving | 2.0 | 4.0 | 57.0 | 19.0 | 3.0 | 10.0 | 350 |  |
|  | Lasagna | 1 serving | 5.5 | 4.5 | 44.0 | 12.0 | 4.0 | 30.0 | 320 |  |
|  | Yoplait yogurt | 6 oz. | 1.0 | 0.5 | 33.0 | 5.0 | 0.0 | 10.0 | 170 |  |
|  | **Total** |  | **56.0** | | **366.0** | **89.0** | **29.0** | **95.0** | **2332** |  |
|  | **% Energy from food** |  | **22%** | | **63%** | **15%** |  |  |  |  |
|  | | | | | | | | | | |
| **Diet B** | Avocado | 120g | 2.5 | 14.3 | 9.6 | 2.4 | 8.2 | 0.0 | 199 | **1.63** |
|  | Pine nuts | 60g | 6.3 | 65.7 | 24.0 | 12.0 | 3.5 | 0.0 | 792 |  |
|  | Heavy whipping cream | 20mL | 5.0 | 3.0 | 0.0 | 0.0 | 0.0 | 30.0 | 72 |  |
|  | Roasted Almonds | 180g | 6.5 | 90.0 | 32.1 | 38.6 | 20.0 | 0.0 | 1151 |  |
|  | **Total** |  | **193.3** | | **65.7** | **53.0** | **31.7** | **30.0** | **2214** |  |
|  | **% Energy from food** |  | **79%** | | **12%** | **9%** |  |  |  |  |
|  | | | | | | | | | | |
| **Diet C** | Smart balance buttery spread | 74g | 10.1 | 35.3 | 0.0 | 0.0 | 0.0 | 0.0 | 410 | **3.81** |
|  | Avocado | 150g | 3.0 | 17.9 | 12.0 | 3.0 | 10.3 | 0.0 | 249 |  |
|  | Heavy whipping cream | 120mL | 30.0 | 18.0 | 0.0 | 0.0 | 0.0 | 180.0 | 432 |  |
|  | Chopped pecans | 60g | 3.7 | 39.1 | 8.5 | 6.4 | 5.9 | 0.0 | 446 |  |
|  | Pine nuts | 40g | 4.1 | 43.8 | 16.0 | 8.0 | 2.3 | 0.0 | 528 |  |
|  | **Total** |  | **205.0** | | **36.5** | **17.4** | **18.5** | **180.0** | **2065** |  |
|  | **% Energy from food** |  | **90%** | | **7%** | **3%** |  |  |  |  |

**Table S7: Detailed diet compositions used in the study for subject #7.**

|  | **Food** | **Amount** | **Total F (g)** | | **Total C (g)** | **Total P (g)** | **Fiber (g)** | **Cholesterol (mg)** | **Total calories (kcal)** | **Ratio of fat: (carb+protein) by mass** |
| --- | --- | --- | --- | --- | --- | --- | --- | --- | --- | --- |
|  |  |  | **Sat. F (g)** | **Unsat. F (g)** |  |  |  |  |  |  |
| **Diet A** | Low fat milk | 16oz. | 4.0 | 0.0 | 26.0 | 16.0 | 0.0 | 24.0 | 204 | **0.14** |
|  | Sesame noodles with vegetables | 1 serving | 0.5 | 2.0 | 55.0 | 10.0 | 7.0 | 0.0 | 280 |  |
|  | Roasted Almonds | 20g | 0.7 | 10.0 | 3.6 | 4.4 | 2.0 | 0.0 | 128 |  |
|  | Lean Cuisine stir fry vegetables and shrimp | 1 serving | 0.5 | 2.0 | 42.0 | 12.0 | 5.0 | 50.0 | 240 |  |
|  | Chicken fried rice | 2 cups | 4.0 | 17.0 | 70.0 | 21.0 | 2.2 | 180.0 | 550 |  |
|  | Chicken Teriyaki | 1 serving | 1.0 | 2.5 | 44.0 | 17.0 | 3.0 | 35.0 | 280 |  |
|  | Gala Apples (small) | 1 | 0.0 | 0.0 | 17.0 | 0.0 | 2.5 | 0.0 | 68 |  |
|  | Mini Truffle cheesecake | ½ serving | 5.5 | 5.0 | 15.0 | 3.0 | 1.5 | 0.0 | 175 |  |
|  | Yoplait yogurt | 6 oz. | 1.0 | 0.5 | 33.0 | 5.0 | 0.0 | 10.0 | 170 |  |
|  | **Total** |  | **56.2** | | **305.6** | **88.4** | **23.2** | **299.0** | **2095** |  |
|  | **% Energy from food** |  | **24%** | | **59%** | **17%** |  |  |  |  |
|  | | | | | | | | | | |
| **Diet B** | Avocado | 150g | 3.0 | 17.9 | 12.0 | 3.0 | 10.3 | 0.0 | 249 | **1.63** |
|  | Pine nuts | 70g | 7.4 | 76.7 | 28.0 | 14.0 | 4.1 | 0.0 | 924 |  |
|  | Roasted Almonds | 120g | 4.3 | 60.0 | 21.4 | 25.7 | 12.9 | 0.0 | 767 |  |
|  | **Total** |  | **169.3** | | **61.4** | **42.7** | **27.3** | **0.0** | **1940** |  |
|  | **% Energy from food** |  | **79%** | | **13%** | **8%** |  |  |  |  |
|  | | | | | | | | | | |
| **Diet C** | Mild sausage patties | 4 patty | 18.3 | 29.7 | 0.0 | 16.0 | 0.0 | 1000.0 | 496 | **3.80** |
|  | Dean’s guacamole dip | 82g | 6.7 | 17.8 | 5.5 | 2.7 | 0.0 | 0.0 | 254 |  |
|  | Heavy whipping cream | 140mL | 35.0 | 21.0 | 0.0 | 0.0 | 0.0 | 210.0 | 504 |  |
|  | Chopped pecans | 40g | 2.6 | 26.1 | 5.7 | 4.3 | 3.9 | 0.0 | 297 |  |
|  | Pine nuts | 23g | 2.5 | 27.5 | 10.0 | 5.0 | 1.8 | 0.0 | 330 |  |
|  | Black coffee | 1 cup | 0.0 | 0.0 | 0.0 | 0.0 | 0.0 | 0.0 | 2 |  |
|  | **Total** |  | **187.2** | | **21.2** | **28.0** | **5.7** | **1210** | **1883** |  |
|  | **% Energy from food** |  | **89%** | | **5%** | **6%** |  |  |  |  |

**Table S8: Detailed diet compositions used in the study for subject #8.**

|  | **Food** | **Amount** | **Total F (g)** | | **Total C (g)** | **Total P (g)** | **Fiber (g)** | **Cholesterol (mg)** | **Total calories (kcal)** | **Ratio of fat: (carb+protein) by mass** |
| --- | --- | --- | --- | --- | --- | --- | --- | --- | --- | --- |
|  |  |  | **Sat. F (g)** | **Unsat. F (g)** |  |  |  |  |  |  |
| **Diet A** | Black coffee + sugar + cream | 2 cups | 1.0 | 1.5 | 20.0 | 2.5 | 0.0 | 5.0 | 113 | **0.15** |
|  | Chicken Fingers Meal | 1 serving | 4.5 | 16.5 | 56.0 | 17.0 | 5.0 | 50.0 | 530 |  |
|  | Sesame Noodles with Vegetables | 1 serving | 0.5 | 1.8 | 55.0 | 10.0 | 7.0 | 0.0 | 280 |  |
|  | Sweet and Sour chicken with rice | 1 serving | 0.5 | 2.5 | 66.0 | 17.0 | 1.0 | 15.0 | 330 |  |
|  | Yoplait yogurt | 6 oz. | 1.0 | 0.5 | 33.0 | 5.0 | 0.0 | 10.0 | 170 |  |
|  | Hershey’s Milk Chocolate | 1 bar | 8.0 | 5.0 | 26.0 | 3.0 | 1.0 | 10.0 | 210 |  |
|  | Vanilla ice Cream | 6 oz. | 4.0 | 3.0 | 31.0 | 5.0 | 0.0 | 50.0 | 210 |  |
|  | **Total** |  | **50.3** | | **287.0** | **59.5** | **14.0** | **140.0** | **1843** |  |
|  | **% Energy from food** |  | **25%** | | **62%** | **13%** |  |  |  |  |
|  | | | | | | | | | | |
| **Diet B** | Roasted Almonds | 120g | 4.3 | 60.0 | 21.4 | 25.7 | 12.9 | 0.0 | 767 | **1.63** |
|  | Pine nuts | 70g | 7.4 | 76.7 | 28.0 | 14.0 | 4.1 | 0.0 | 924 |  |
|  | Avocado | 120g | 2.5 | 14.3 | 9.6 | 2.4 | 8.2 | 0.0 | 199 |  |
|  | Black coffee | 1 cup | 0.0 | 0.0 | 0.0 | 0.0 | 0.0 | 0.0 | 2 |  |
|  | **Total** |  | **165.2** | | **59.0** | **42.1** | **25.2** | **0.0** | **1892** |  |
|  | **% Energy from food** |  | **79%** | | **12%** | **9%** |  |  |  |  |
|  | | | | | | | | | | |
| **Diet C** | Mild sausage patties | 2 patty | 9.2 | 14.8 | 0.0 | 8.0 | 0.0 | 500.0 | 248 | **3.80** |
|  | Dean’s guacamole dip | 82g | 6.7 | 17.8 | 5.5 | 2.7 | 0.0 | 0.0 | 254 |  |
|  | Heavy whipping cream | 140mL | 35.0 | 21.0 | 0.0 | 0.0 | 0.0 | 210.0 | 504 |  |
|  | Chopped pecans | 45g | 2.9 | 29.3 | 6.4 | 4.8 | 4.4 | 0.0 | 334 |  |
|  | Pine nuts | 30g | 3.1 | 32.9 | 12.0 | 6.0 | 1.8 | 0.0 | 396 |  |
|  | Black coffee | 1 cup | 0.0 | 0.0 | 0.0 | 0.0 | 0.0 | 0.0 | 2 |  |
|  | **Total** |  | **172.7** | | **23.9** | **21.5** | **6.2** | **710.0** | **1738** |  |
|  | **% Energy from food** |  | **89%** | | **6%** | **5%** |  |  |  |  |

**Table S9: Detailed diet compositions used in the study for subject #9.**

|  | **Food** | **Amount** | **Total F (g)** | | **Total C (g)** | **Total P (g)** | **Fiber (g)** | **Cholesterol (mg)** | **Total calories (kcal)** | **Ratio of fat: (carb+protein) by mass** |
| --- | --- | --- | --- | --- | --- | --- | --- | --- | --- | --- |
|  |  |  | **Sat. F (g)** | **Unsat. F (g)** |  |  |  |  |  |  |
| **Diet A** | Pasta Primavera | 1 serving | 2.0 | 2.0 | 41.0 | 11.0 | 4.0 | 5.0 | 250 | **0.12** |
|  | Sweet & Sour Chicken & Rice | 1 serving | 0.5 | 2.5 | 66.0 | 10.0 | 1.0 | 15.0 | 330 |  |
|  | Mac and Cheese | 1 serving | 1.0 | 1.0 | 50.0 | 11.0 | 2.0 | 5.0 | 260 |  |
|  | Fettuccine Alfredo with Chicken Broccoli | 1 serving | 5.0 | 4.0 | 36.0 | 14.0 | 2.0 | 40.0 | 280 |  |
|  | Cheese Ravioli | 1 serving | 2.5 | 2.5 | 38.0 | 11.0 | 3.0 | 45.0 | 240 |  |
|  | Fettuccine Alfredo | 1 serving | 6.0 | 4.0 | 45.0 | 10.0 | 2.0 | 30.0 | 310 |  |
|  | Blueberry low fat yogurt | 6oz. | 1.0 | 0.5 | 33.0 | 6.0 | 0.0 | 10.0 | 170 |  |
|  | Raspberry low fat yogurt | 6oz. | 1.0 | 0.5 | 33.0 | 6.0 | 0.0 | 10.0 | 170 |  |
|  | Gala apples | 3 | 0.0 | 0.0 | 51.0 | 0.0 | 7.5 | 0.0 | 204 |  |
|  | Banana | 3 | 0.3 | 0.9 | 81.0 | 3.9 | 9.3 | 0.0 | 341 |  |
|  | Pine nuts | 23g | 2.5 | 27.5 | 10.0 | 5.0 | 1.8 | 0.0 | 330 |  |
|  | Vanilla ice Cream | 6 oz. | 4.0 | 3.0 | 31.0 | 5.0 | 0.0 | 50.0 | 210 |  |
|  | **Total** |  | **74.2** | | **515.0** | **92.9** | **32.6** | **210.0** | **3095** |  |
|  | **% Energy from food** |  | **22%** | | **66%** | **12%** |  |  |  |  |
|  | | | | | | | | | | |
| **Diet B** | Avocado | 200g | 4.2 | 23.8 | 16.0 | 4.0 | 13.7 | 0.0 | 332 | **1.63** |
|  | Pine nuts | 100g | 10.5 | 109.5 | 40.0 | 20.0 | 5.8 | 0.0 | 1320 |  |
|  | Roasted Almonds | 165g | 5.9 | 82.5 | 29.4 | 35.3 | 17.7 | 0.0 | 1055 |  |
|  | **Total** |  | **236.4** | | **85.4** | **59.3** | **37.2** | **0.0** | **2707** |  |
|  | **% Energy from food** |  | **79%** | | **13%** | **8%** |  |  |  |  |
|  | | | | | | | | | | |
| **Diet C** | Mild sausage patties | 4 patty | 18.3 | 29.7 | 0.0 | 16.0 | 0.0 | 1000.0 | 496 | **3.80** |
|  | Avocado | 75g | 1.6 | 8.9 | 6.0 | 1.5 | 5.2 | 0.0 | 125 |  |
|  | Heavy whipping cream | 220mL | 55.0 | 33.0 | 0.0 | 0.0 | 0.0 | 300.0 | 792 |  |
|  | Chopped pecans | 115g | 7.3 | 74.9 | 16.3 | 12.3 | 11.3 | 0.0 | 854 |  |
|  | Pine nuts | 28g | 2.9 | 30.7 | 11.2 | 5.6 | 1.6 | 0.0 | 370 |  |
|  | Black coffee | 1 cup | 0.0 | 0.0 | 0.0 | 0.0 | 0.0 | 0.0 | 2 |  |
|  | **Total** |  | **262.3** | | **33.5** | **35.4** | **18.1** | **1300** | **2639** |  |
|  | **% Energy from food** |  | **90%** | | **5%** | **5%** |  |  |  |  |

**Table S10: Detailed diet compositions used in the study for subject #10.**

|  | **Food** | **Amount** | **Total F (g)** | | **Total C (g)** | **Total P (g)** | **Fiber (g)** | **Cholesterol (mg)** | **Total calories (kcal)** | **Ratio of fat: (carb+protein) by mass** |
| --- | --- | --- | --- | --- | --- | --- | --- | --- | --- | --- |
|  |  |  | **Sat. F (g)** | **Unsat. F (g)** |  |  |  |  |  |  |
| **Diet A** | Black coffee + sugar + cream | 2 cups | 1.0 | 1.5 | 20.0 | 2.5 | 0.0 | 5.0 | 113 | **0.17** |
|  | Chicken Alfredo with Fettuccine | 1 serving | 9.0 | 8.0 | 49.0 | 29.0 | 5.0 | 100.0 | 470 |  |
|  | Sesame Noodles with Vegetables | 1 serving | 0.5 | 1.8 | 55.0 | 10.0 | 7.0 | 0.0 | 280 |  |
|  | Sweet & Sour Chicken & Rice | 1 serving | 2.5 | 11.5 | 56.0 | 10.0 | 3.0 | 20.0 | 390 |  |
|  | Beef Steak & Noodles | 1 serving | 5.0 | 9.0 | 51.0 | 33.0 | 4.0 | 105.0 | 460 |  |
|  | Gala Apples (small) | 2 | 0.0 | 0.0 | 33.0 | 0.0 | 5.0 | 0.0 | 130 |  |
|  | Yoplait yogurt | 6 oz. | 1.0 | 0.5 | 33.0 | 5.0 | 0.0 | 10.0 | 170 |  |
|  | Pine nuts | 15g | 1.4 | 16.6 | 6.0 | 3.0 | 1.0 | 0.0 | 198 |  |
|  | **Total** |  | **69.3** | | **304.0** | **92.5** | **25.0** | **240.0** | **2211** |  |
|  | **% Energy from food** |  | **28%** | | **55%** | **17%** |  |  |  |  |
|  | | | | | | | | | | |
| **Diet B** | Pine nuts | 67g | 7.0 | 73.4 | 26.8 | 13.4 | 3.9 | 0.0 | 884 | **1.63** |
|  | Avocado | 120g | 2.5 | 14.3 | 9.6 | 2.4 | 8.2 | 0.0 | 199 |  |
|  | Roasted Almonds | 120g | 4.3 | 60.0 | 21.4 | 25.7 | 12.9 | 0.0 | 767 |  |
|  | **Total** |  | **161.5** | | **57.8** | **41.5** | **25.0** | **0.0** | **1850** |  |
|  | **% Energy from food** |  | **79%** | | **12%** | **9%** |  |  |  |  |
|  | | | | | | | | | | |
| **Diet C** | Mild sausage patties | 4 patty | 18.3 | 29.7 | 0.0 | 16.0 | 0.0 | 1000.0 | 496 | **3.80** |
|  | Avocado | 60g | 1.2 | 7.2 | 4.8 | 1.2 | 4.1 | 0.0 | 100 |  |
|  | Heavy whipping cream | 150mL | 37.5 | 22.5 | 0.0 | 0.0 | 0.0 | 225 | 540 |  |
|  | Chopped pecans | 70g | 4.6 | 45.4 | 10.0 | 7.5 | 7.3 | 0.0 | 520 |  |
|  | Pine nuts | 15g | 1.4 | 16.6 | 6.0 | 3.0 | 1.0 | 0.0 | 198 |  |
|  | **Total** |  | **184.4** | | **20.8** | **27.7** | **12.4** | **1225** | **1854** |  |
|  | **% Energy from food** |  | **90%** | | **4%** | **6%** |  |  |  |  |

**Table S11: Detailed diet compositions used in the study for subject #11.**

|  | **Food** | **Amount** | **Total F (g)** | | **Total C (g)** | **Total P (g)** | **Fiber (g)** | **Cholesterol (mg)** | **Total calories (kcal)** | **Ratio of fat: (carb+protein) by mass** |
| --- | --- | --- | --- | --- | --- | --- | --- | --- | --- | --- |
|  |  |  | **Sat. F (g)** | **Unsat. F (g)** |  |  |  |  |  |  |
| **Diet A** | Large egg | 3 | 5.5 | 9.5 | 4.0 | 20.0 | 0.0 | 560.0 | 231 | **0.25** |
|  | Olive oil | 3 tbsp. | 6.3 | 37.6 | 0.0 | 0.0 | 0.0 | 0.0 | 395 |  |
|  | Spinach | 3.5 oz. | 0.0 | 0.2 | 3.0 | 2.4 | 1.7 | 0.0 | 23 |  |
|  | Carrot (medium) | 3 | 0.0 | 0.3 | 18.0 | 1.8 | 5.4 | 0.0 | 82 |  |
|  | Tofu | 1/5 block | 0.7 | 3.5 | 1.8 | 7.2 | 0.3 | 0.0 | 74 |  |
|  | Whole grain Bread | 3.5 slide | 1.0 | 2.5 | 126.0 | 28.0 | 5.3 | 0.0 | 648 |  |
|  | Jelly | 3 tbsp. | 0.0 | 0.0 | 39.0 | 0.0 | 3.0 | 0.0 | 156 |  |
|  | Ice cream sandwich | 1 sandwich | 3.5 | 7.5 | 52.0 | 6.0 | 0.0 | 30.0 | 331 |  |
|  | **Total** |  | **78.1** | | **243.8** | **65.4** | **15.7** | **590.0** | **1940** |  |
|  | **% Energy from food** |  | **36%** | | **50%** | **14%** |  |  |  |  |
|  | | | | | | | | | | |
| **Diet B** | Avocado | 120g | 2.5 | 14.3 | 9.6 | 2.4 | 8.2 | 0.0 | 199 | **1.63** |
|  | Pine nuts | 70g | 7.4 | 76.7 | 28.0 | 14.0 | 4.1 | 0.0 | 924 |  |
|  | Roasted Almonds | 120g | 4.3 | 60.0 | 21.4 | 25.7 | 12.9 | 0.0 | 767 |  |
|  | **Total** |  | **165.2** | | **59.0** | **42.1** | **25.2** | **0.0** | **1890** |  |
|  | **% Energy from food** |  | **79%** | | **12%** | **9%** |  |  |  |  |
|  | | | | | | | | | | |
| **Diet C** | Mild sausage patties | 2 patty | 9.2 | 14.8 | 0.0 | 8.0 | 0.0 | 500.0 | 248 | **3.80** |
|  | Avocado | 40g | 0.8 | 4.8 | 3.2 | 0.7 | 4.4 | 0.0 | 66 |  |
|  | Heavy whipping cream | 150mL | 37.5 | 22.5 | 0.0 | 0.0 | 0.0 | 225 | 540 |  |
|  | Chopped pecans | 65g | 4.0 | 42.4 | 9.3 | 7.0 | 4.2 | 0.0 | 483 |  |
|  | Pine nuts | 35g | 3.3 | 38.7 | 14.0 | 7.0 | 2.3 | 0.0 | 462 |  |
|  | Smart balance buttery spread | 15g | 2.9 | 6.3 | 0.0 | 0.0 | 0.0 | 0.0 | 83 |  |
|  | Black coffee | 1 cup | 0.0 | 0.0 | 0.0 | 0.0 | 0.0 | 0.0 | 2 |  |
|  | **Total** |  | **187.2** | | **26.5** | **22.7** | **10.9** | **732** | **1884** |  |
|  | **% Energy from food** |  | **90%** | | **6%** | **4%** |  |  |  |  |
